# Physiologic Features of High Altitudes

**Published:** 1912-10-15

**Authors:** 


### PHYSIOLOGIC FEATURES OF HIGH ALTITUDES.

There are manifold relations between climate and man which can justly lay claim to scientific interest. Some of them have agricultural or anthropologic bearings, whereas the physician is especially concerned with those features which relate to hygiene or therapy. The underlying problems have been studied in various ways in the past, either by observation under the conditions which actually obtain in nature, or in the laboratory where an attempt is made to imitate and experimentally control individual factors such as temperature, humidity and barometric pressure. Within the past decade investigation in each of these directions has received a noteworthy impetus, so that there is available at present a considerable body of useful scientific literature relating directly to the subject of climate. In a way, laboratory studies like those conducted in the respiration chamber have served to supplement or confirm the evidence collected in the open air.

As happens not infrequently in scientific work, the improvement in apparatus and the refinement of experimental technic, along with an accumulation of healthy criticism, have modified some of the earlier views regarding the effects of certain climatic factors. It may be helpful to take a retrospect of the advance in knowledge which a decade of investigation has brought in respect to the physiology of altitude.[1] A few years ago it was confidently stated that removal to higher altitudes was attended with a decided increase in the number of red corpuscles and the percentage of hemoglobin in the blood. This result was looked on as a compensatory response on the part of the organism, whereby the possible inability of the body to satisfy its oxygen needs in the presence of a lowered barometric pressure was averted. With improved technic in hematologic work, whereby especially the more rapid evaporation of blood samples at higher altitudes is avoided, the tendency has been for the earlier reported extreme increases in the oxygen-carrying blood constituents to disappear. The reports of more recent years such as those made by Burker and his

1For a very recent review of this subject see Cohnheim, O.: Physiologie des Alpinismus, II Ergebn. d. Physiol., 1912, xii, 628; also Anglo-American Expedition to Pike's Peak, The Journal A. M. A., Aug. 10, 1912, p. 449.

colleagues[2], show at best only a comparatively small increase amounting to 4 or 5 per cent. at altitudes of five or six thousand feet. The same moderate results have likewise been noted lately for much higher altitudes. There is no displacement of the usual relations between the number of corpuscles and the content of homoglobin and iron in the blood, contrary to the conditions formerly alleged to exist at lowered barometric pressure. Cohnheim has essayed to attribute to a concentration of the blood at high altitudes the almost insignificant increases which have undeniably been found by competent observers. The climate is always dry and evaporation proceeds rapidly. As a result individuals lose water more readily than at lower levels, and the losses become more pronounced and conspicuous in smaller animals, such as those which commonly serve the purposes of experiment. If this explanation is tenable, the increases in corpuscles or hemoglobin content occasionally found are in no wise the expression of a lack of oxygen; they are rather the outcome of the increased evaporation under the newer conditions of climate. Thereby one reputed response of the organism is no longer tenable as a therapeutic hypothesis.

The influence of altitude on respiration has, of course always been the subject of speculation, especially in its relation to mountain sickness. Two factors come into play. As the altitude increases, a lowered tension of oxygen in the alveolar air results, and there is a diminished tension of carbon dioxid. The rate of respiration may be variously influenced in different circumstances; but the depth of respiration is almost invariably increased. This, of itself, not only facilitates the oxygen supply, but also increases the elimination of carbon dioxid. These effects are reflected in the content of the two gases in the blood. Since Haldane's classic investigations on the regulation of respiration by the carbon dioxid tension in the blood it has

2See editorial, Altitude and Blood-Corpuscles, THE JOURNAL A. M. A., February 3, p. 344.

become apparent why respiration may become so markedly modified under these conditions. Mosso and his followers were always inclined to attribute to the acapnia a very important role in the physiologic disturbances noted in high altitudes. Undoubtedly acapnia is a factor to be reckoned with, but the primary and fundamental feature is the lowered oxygen pressure to which the other consequences are in reality secondary.

Whether the pulmonary exchange of gases proceeds according to well-known physical laws at high altitudes, or there is an active secretion of oxygen by the pulmonary epithelium into the blood, as has lately been maintained, remains to be determined.[3] In addition to these purely respiratory influences there is evidence of an increased combustion in the body under conditions of lowered oxygen tension, and there are undeniable influences on nitrogenous metabolism. Effects of this type still await an explanation. Unquestionably very high altitudes—and perhaps those moderately high—are detrimental to human health, but the great body of clinical climatologic literature still needs to be sifted and studied in the light of modern physiology before an adequate understanding of reputed and unquestioned curative virtues of mountain resorts can be attained.—Editorial in Sept. 12, 1912, *A. M. A. Journal.*

